# BdAreA regulates nitrogen metabolism, stress response, and virulence in *Botryosphaeria dothidea*

**DOI:** 10.1007/s44297-026-00065-8

**Published:** 2026-01-22

**Authors:** Dewan Zhang, Chunmei Han, Na Liu, Weichao Ren, Sen Lian, Baohua Li, Caixia Wang

**Affiliations:** 1 Shandong Engineering Research Center for Environment-Friendly Agricultural Pest Management, Qingdao Engineering Research Center of Precise Control of Fruit and Vegetable Pests, College of Plant Health and Medicine, Qingdao Agricultural University, Qingdao, Shandong China; 2 Shandong Provincial Key Laboratory of Microbial Resource Exploration and Innovative Utilization, Qingdao Agricultural University, Qingdao, Shandong China

**Keywords:** *Botryosphaeria dothidea*, Stress response, Virulence, Nitrogen metabolism, MAPK

## Abstract

The GATA-family transcription factor AreA is a central regulator of nitrogen metabolism in filamentous fungi, and it exhibits significant functional divergence among different fungal species. In this study, using *Botryoschaeria dothidea* (*B. dothidea*), the causal agent of apple ring rot, as the target organism, we systematically characterized the pleiotropic functions of *BdAreA* (GATA-type zinc-finger transcription factor AreA of *B. dothidea*) through targeted gene knockout. Phenotypic analysis showed that conidiation production was inhibited in Δ*BdAreA*, and its pathogenicity was significantly attenuated. In addition, the mutant exhibited sensitivity to both hydrogen peroxide and phytoalexins. Notably, *BdAreA* specifically regulated the utilization of nitrate (NO₃⁻), and combined real-time quantitative PCR (RT-qPCR) and enzyme activity assays confirmed that it positively regulates both the transcriptional level and catalytic activity of nitrate reductase (BdNR) and nitrite reductase (BdNIR) of *B. dothidea*. More importantly, this study revealed for the first time that *BdAreA* negatively regulates the expression of BdHog1 and affects its phosphorylation level. Our results demonstrate that *BdAreA* is a master regulator controlling growth, development, pathogenicity, stress response, and nitrogen metabolism in *B. dothidea* while also providing important theoretical insights into the link between nitrogen metabolic pathways and mitogen-activated protein kinase (MAPK) signaling cascades.

**Supplementary Information:**

The online version contains supplementary material available at 10.1007/s44297-026-00065-8.

## Introduction

Apple ring rot, caused by the fungal pathogen *Botryosphaeria dothidea*, represents one of the most devastating diseases in China's major apple-producing regions [1, 2]. This pathogen systemically infects both branches and fruits of apple trees, causing severe damage during orchard growth and postharvest storage, resulting in substantial annual economic losses [3]. Notably, the predominant cultivar 'Fuji' exhibits high susceptibility to this disease, making chemical control the primary management strategy [4]. However, the prolonged exclusive use of traditional fungicides such as benzimidazoles has led to widespread resistance in field populations [5]. Therefore, elucidating the molecular regulatory networks governing vegetative growth, pathogenicity, and stress responses in *B. dothidea* will not only identify potential targets for novel fungicides but also guide the development of sustainable integrated management strategies.

Precise regulation of gene expression is fundamental to biological processes, with transcriptional control playing a central role. In filamentous fungi, the nitrogen metabolite repression (NMR) system, mediated by GATA-type zinc-finger transcription factors, serves as the key mechanism regulating nitrogen source utilization. The core component of this system, AreA and its homologs, act as master regulators of nitrogen metabolism by activating genes involved in non-preferred nitrogen source assimilation, ensuring fungal adaptation to varying nitrogen environments [6].

In-depth studies reveal that AreA displays both conserved and divergent regulatory features across filamentous fungi. In *Aspergillus nidulans*, AreA deletion mutants can only utilize ammonium as a sole nitrogen source [7], whereas in *Fusarium fujikuroi*, AreA specifically regulates nitrate assimilation and urea metabolism [8]. In *F. graminearum* and *F. oxysporum*, AreA exerts stronger control over nitrate utilization [9, 10]. Notably, the functional repertoire of AreA extends beyond nitrogen metabolism: in *F. graminearum,* it directly controls deoxynivalenol (DON) mycotoxin biosynthesis [9, 11]; in *F. oxysporum* and *T. atroviride,* it orchestrates pathogenicity by regulating virulence factors, including siderophore biosynthesis [12–14], while in *Aureobasidium* species, its homolog Gat1 governs polymalic acid production in *A. pullulans* [15], and AreA itself mediates pullulan biosynthesis in *A. melanogenum* [16]. However, comparative genomic analyses demonstrate species-specific roles in pathogenesis—the AreA homolog in *Cladosporium fulvum* only partially contributes to virulence [17], while in *Magnaporthe oryzae*, it shows no association with pathogenicity [18]. These findings highlight the functional diversification of AreA regulatory networks during the evolution of filamentous fungi.

This study investigated the biological functions of the GATA transcription factor BdAreA in *B. dothidea*. The results demonstrate that *BdAreA* plays critical roles in asexual development, plant infection, stress responses, and nitrogen metabolism. Significantly, our study revealed a previously unrecognized connection between this GATA transcription factor and the MAPK signaling cascade. These findings provide a theoretical foundation for developing targeted fungicides to control apple ring rot.

## Results

### Nitrogen deficiency induces upregulation of *BdAreA*

**Fig. 1:**
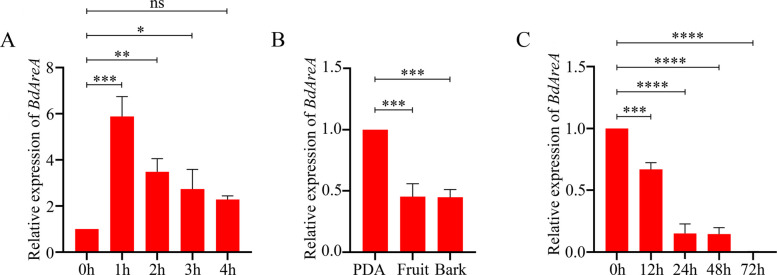
Measurement of *BdAreA* gene expression in the wild-type strain. **A**: Gene expression levels in mycelia under nitrogen starvation at different time points; **B**: Gene expression levels in mycelia grown on different culture media; **C**: Gene expression levels during different stages of mycelial infection. The error bars represent the standard deviation of three independent experiments; asterisks indicate significant differences between groups ("ns" indicates no significant difference; *P < 0.05; **P < 0.01; ***P < 0.001; ****P < 0.0001)

Our phylogenetic analysis identified the *B. dothidea* zinc-finger GATA protein (KAF4304195) as a member of the AreA subfamily, which was subsequently designated BdAreA (Fig. S1). This transcription factor displays remarkable evolutionary conservation, implying critical conserved functions across fungal species. Expression analysis demonstrated complex regulation of *BdAreA* under various conditions. Under nitrogen deprivation, *BdAreA* expression surged rapidly, reaching a 5.9-fold induction peak within 1 h (Fig. 1A). In striking contrast, growth on natural substrates (fruit and bark media) suppressed *BdAreA* expression to 48% and 39% of PDA control levels, respectively (Fig. 1B). During host infection, *BdAreA* displayed progressive transcriptional silencing, with significant downregulation by 12 hpi, stabilization at 24 hpi, and near-complete suppression by 72 hpi (Fig. 1C). These diametrically opposed expression profiles suggest that *BdAreA* serves dichotomous regulatory functions—upregulating during nitrogen limitation while being actively suppressed during pathogenic development, although the molecular mechanisms governing this switch remain to be fully elucidated.

### BdAreA regulates mycelial growth in *B. dothidea*

**Fig.2:**
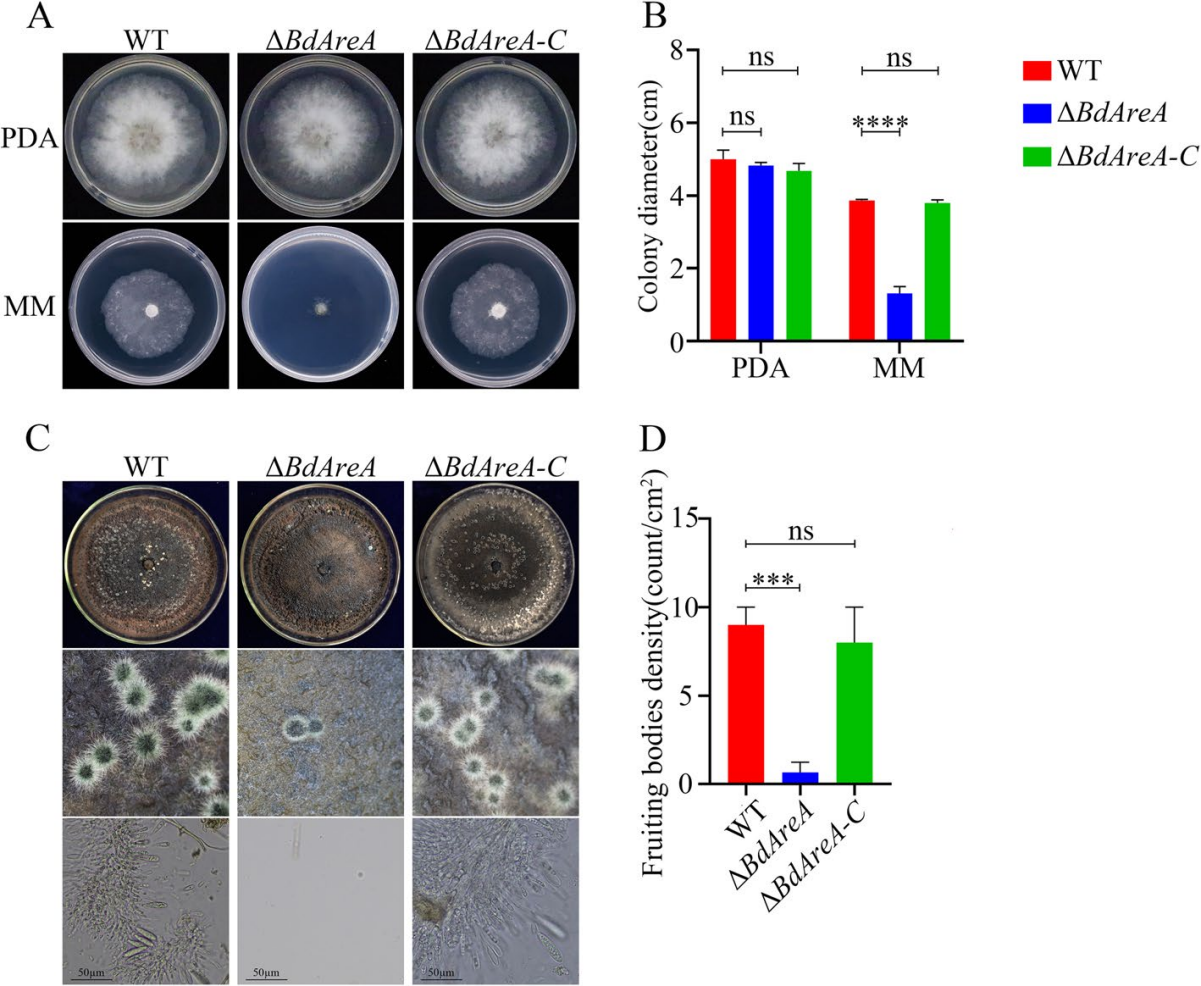
Colony morphology, vegetative growth and sporulation of WT, Δ*BdAreA* and Δ*BdAreA-C*. **A**: Colony morphology of different strains grown on PDA and MM at 25 °C for 2 days. **B**: Average colony diameter of incubation of each strain after 2 days of culture on PDA and MM at 25 °C. **C**: The morphology of fruiting bodies and conidia formed by individual strains on carrot culture medium; **D**: Statistics on the number of subentities per square centimeter. The error bars represent the standard deviation of three independent experiments; asterisks indicate significant differences between groups ("ns" indicates no significant difference; ***P < 0.001)

We examined the functional contribution of *BdAreA* to mycelial growth by comparing colony development of the wild-type strain (WT), knockout mutant Δ*BdAreA*, and complemented strain Δ*BdAreA-C* (Fig. S2) on both nutrient-rich (PDA) and minimal (MM) media (Fig. 2A, B). On PDA medium, all strains exhibited comparable growth patterns, characterized by dense aerial mycelium and similar growth rates. In contrast, Δ*BdAreA* was severely compromised on MM medium, producing only sparse and underdeveloped mycelia with a markedly reduced growth rate, which stood in sharp contrast to the robust growth of WT and Δ*BdAreA-C*. This phenotype establishes that *BdAreA* is indispensable for normal fungal growth under nutrient-limiting conditions, revealing its critical role in supporting mycelial development during the vegetative growth phase.

### *BdAreA* plays a critical role in asexual reproduction

Conidia formation is a critical determinant for the epidemic spread of *B. dothidea*. To evaluate the impact of *BdAreA* on asexual reproduction, we conducted black light-induced sporulation assays on carrot agar. After 7 days of induction, microscopic examination revealed notable differences among the strains: WT produced abundant fruiting bodies (10–11 structures/cm^2^) containing numerous conidia, whereas Δ*BdAreA* formed only sparse fruiting bodies, with no conidia detected within them (Fig. 2C, D). Δ*BdAreA-C* restored pycnidial formation and conidiation capacity to levels comparable to those of the wild-type strain. These results indicate that *BdAreA* is involved in and plays a role during asexual reproduction of *B. dothidea* on carrot agar, thereby indirectly linking this transcription factor to the pathogen's epidemic potential.

### BdAreA is important for pathogenesis

**Fig. 3:**
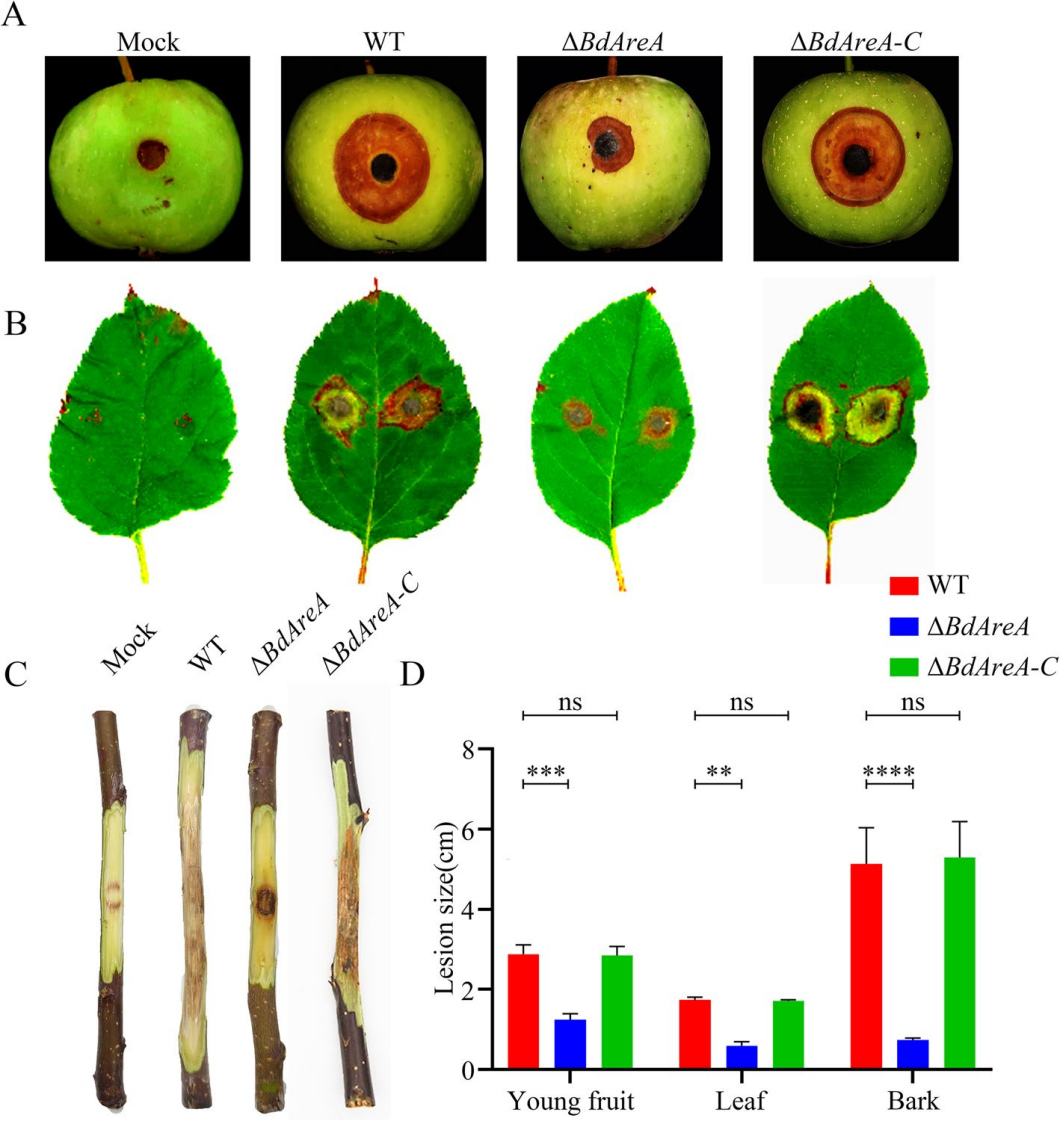
Effects of *BdAreA* deletion on the pathogenicity of *B. dothidea.* **A**, **B** and **C**: Apple fruits, apple leaves and twigs inoculated with different strains; **D**: Lesion size on apple leaves and twigs after inoculation. The error bars represent the standard deviation of three independent experiments; asterisks indicate significant differences between groups ("ns" indicates no significant difference; **P < 0.01; ***P < 0.001; ****P < 0.0001)

We systematically evaluated the virulence contribution of *BdAreA* through multi-tissue infection assays using 'Fuji' apple fruits, one-year-old twigs, and leaves inoculated with WT, Δ*BdAreA*, Δ*BdAreA-C*, and PDA control (Fig. 3). Following incubation at 25 °C for 3–7 days, quantitative lesion measurements revealed striking pathogenicity differences. WT induced characteristic ring rot symptoms with mean lesion diameters of 2.95 cm (fruits) and 0.75 cm (leaves) and twig lesion lengths of 5.15 cm—measurements that were fully restored in Δ*BdAreA-C*. In marked contrast, Δ*BdAreA* exhibited severely attenuated virulence, producing significantly smaller lesions (1.25 cm in fruits, 0.6 cm in twigs, and 0.75 cm in leaves; p < 0.05 versus the wild-type strain). This consistent reduction in lesion development across all tested plant tissues provides compelling evidence that *BdAreA* functions as a central virulence determinant in *B. dothidea*, essential for full pathogenic capacity during host infection.

### *BdAreA* regulates oxidative stress and phytoalexin responses in *B. dothidea*

**Fig. 4:**
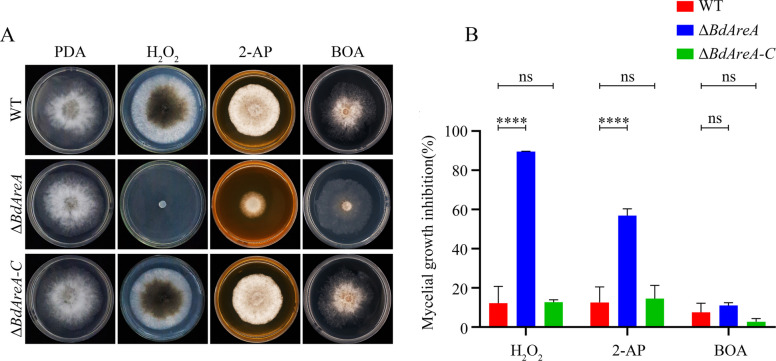
Determination of sensitivity to stress factors of Δ*BdAreA.* **A:** Sensitivity assays of the strains to H_2_O_2_ and phytoalexins (2-AP and BOA); **B**: The mycelial inhibition rate of each strain. The error bars represent the standard deviation of three independent experiments; asterisks indicate significant differences between groups ("ns" indicates no significant difference; *P < 0.05; ****P < 0.0001)

Fungal pathogens employ sophisticated stress response systems to counteract environmental challenges and host defense compounds. To evaluate the role of *BdAreA* in stress adaptation, we subjected WT, Δ*BdAreA*, and Δ*BdAreA-C* to various stressors representing critical defense barriers (Fig. 4). Although osmotic stress (sorbitol, NaCl, KCl), cell wall stress (Congo red), and membrane stress (SDS) exerted similar inhibitory effects on all strains tested, striking phenotypic variations were observed when challenged with oxidative and phytoalexin stresses. Δ*BdAreA* displayed extreme oxidative stress sensitivity, with 96% growth inhibition under H₂O₂ treatment. Phytoalexin exposure revealed compound-specific vulnerabilities: 2-aminoacetophenone (2-AP) caused 54.3% growth suppression, while benzoxazolin-2-one (BOA), although not affecting radial expansion, severely impaired aerial hyphal development. The complemented strain restored WT stress tolerance in all cases, confirming *BdAreA*'s specific role in mediating oxidative defense and phytoalexin detoxification pathways rather than general stress responses. Furthermore, to determine whether the *BdAreA* deletion mutant confers susceptibility to other oxidants, we further assessed its sensitivity to tert-butyl hydroperoxide (t-BHP) and menadione. As shown in Fig. S5, Δ*BdAreA* exhibited a significantly reduced growth rate when exposed to both compounds. These results indicate that *BdAreA* is involved in a broad oxidative stress response, not merely specific to H₂O₂, although the mutant demonstrates heightened sensitivity to the latter. These results position *BdAreA* as a key regulator of *B. dothidea*'s capacity to overcome two critical host defense mechanisms: reactive oxygen species bursts and antimicrobial phytoalexin production. The differential sensitivity patterns suggest that *BdAreA* may coordinate distinct molecular pathways for oxidative stress protection versus secondary metabolite detoxification.

### Nitrate supplementation does not rescue the growth defect in *ΔBdAreA*

**Fig. 5:**
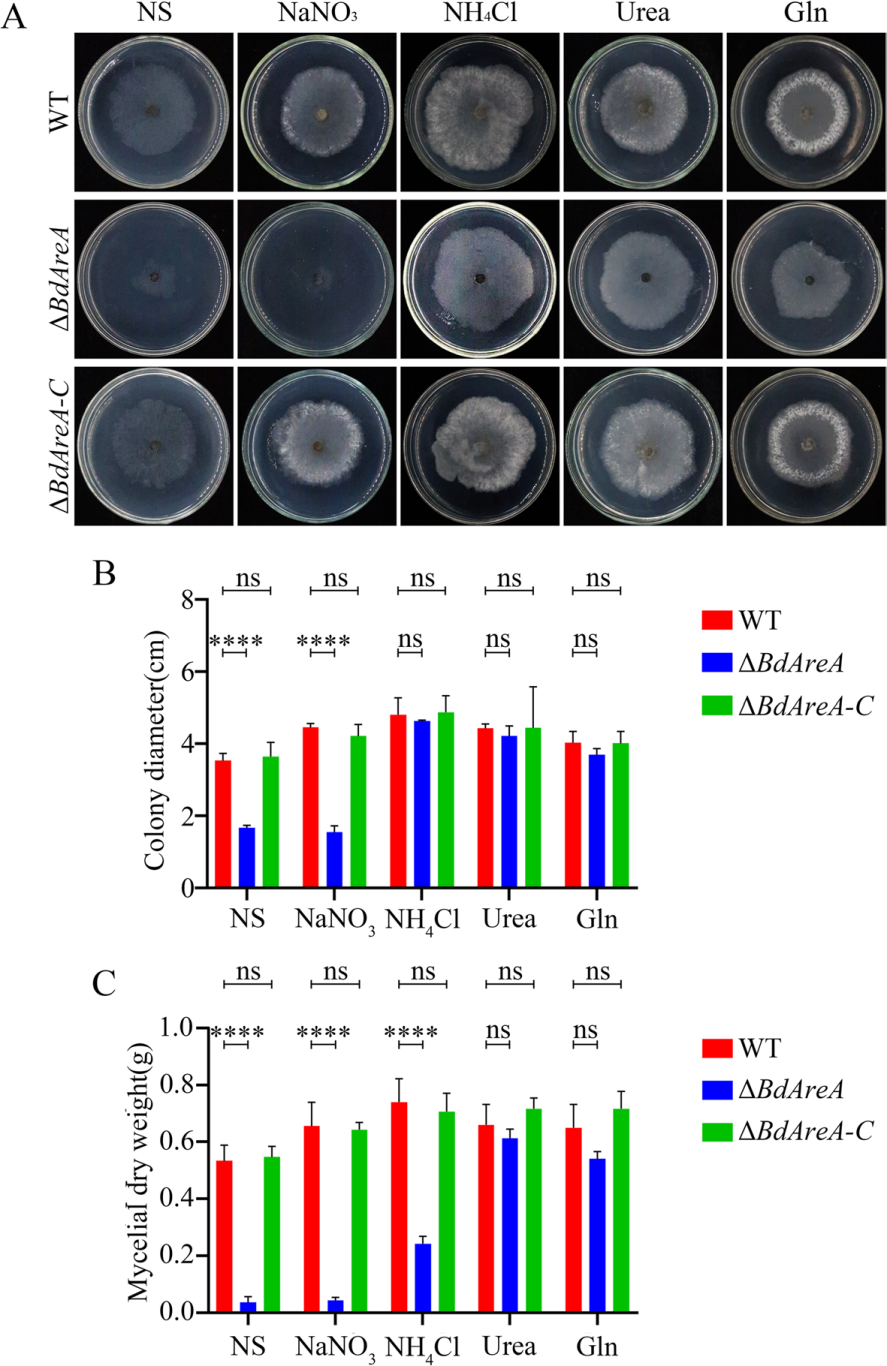
Colony morphology, diameter and mycelial dry weight of different strains. A: Colony phenotypes of different strains cultured at 25 °C in the dark for 3 days on Czapek's medium containing NaNO3, NH4Cl, glutamine, or urea as the sole nitrogen source (10 mmol L.^−1^) as well as nitrogen-free Czapek's medium (NS). **B**: Mycelial diameter of each strain cultured on different solid media at 25 °C for 3 days. **C:** Mycelial dry weight of each strain cultured in different liquid media at 25 °C with 180 rpm shaking for 5 days. The error bars represent the standard deviation of three independent experiments; asterisks indicate significant differences between groups ("ns" indicates no significant difference; ****P < 0.0001)

The capacity of fungi to utilize diverse nitrogen sources is critical for their environmental adaptation, with ammonium and glutamine serving as preferred nutrients due to their direct assimilation pathways. In *F. graminearum*, it has been reported that *AreA* deletion impairs the fungal ability to utilize different nitrogen sources [9]. Consistent with this regulatory role, our investigation of *BdAreA* revealed that Δ*BdAreA* exhibited normal growth on nutrient-rich PDA medium but failed to grow on MM medium with defined nitrogen sources. This observation prompted further analysis using nitrogen-defined Czapek's media. Growth assessment revealed a graded response profile (Fig. 5): the mutant showed absolute growth arrest under nitrogen starvation but achieved full recovery with urea or glutamine supplementation. While NH₄Cl partially restored growth, biomass accumulation remained significantly impaired, and NaNO₃ proved completely non-utilizable. These findings position *BdAreA* as a central regulator coordinating multiple nitrogen assimilation systems, demonstrating its indispensable role in both nitrate utilization and efficient ammonium metabolism. The distinct phenotypic responses to different nitrogen compounds suggest that *BdAreA* mediates complex regulatory networks involving nitrate pathway activation, ammonium uptake optimization, and potential metabolic cross-talk, advancing our understanding of nitrogen source prioritization mechanisms in phytopathogenic fungi.

### *BdAreA* positively regulates the expression and activity of nitrate and nitrite reductases

**Fig. 6:**
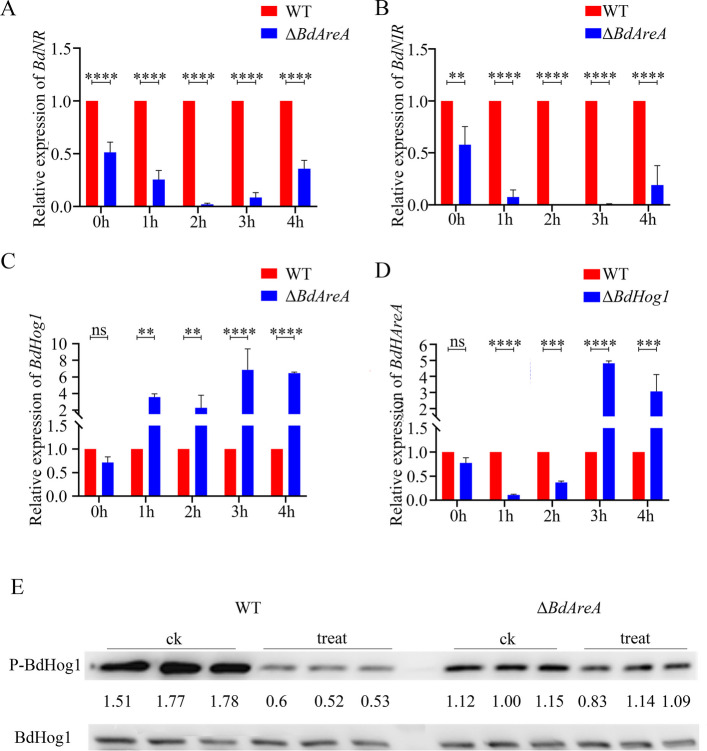
Analysis of gene expression levels and Western blot analysis. Gene expression levels of *BdNR* (**A**), *BdNIR* (**B**), *BdHog1* (**C**) and *BdAreA* (**D**) in mycelia under nitrogen starvation at different time points; **E**: Phosphorylation of BdHog1 treated with nitrogen starvation for 1 h. The error bars represent the standard deviation of three independent experiments; asterisks indicate significant differences between groups ("ns" indicates no significant difference; ****P < 0.0001)

Building on established evidence that Δ*BdAreA* is defective in nitrate assimilation—a process fundamentally dependent on nitrate reductase (NR) and nitrite reductase (NIR) activity—we sought to determine whether this phenotype stems from impaired regulation of *BdNR* and *BdNIR*. Our time-course expression analysis under nitrogen starvation revealed striking transcriptional dysregulation in the mutant (Fig. 6A,B). While basal expression of both genes in the mutant was already reduced to 50% of WT levels under normal conditions, nitrogen starvation induced a dramatic divergence: WT showed characteristic induction peaks, whereas the mutant exhibited progressive suppression, reaching minimal expression levels of 1.5% (BdNR) and 0.28% (BdNIR) relative to WT at the 2 h timepoint before partial recovery. This is consistent with our transcriptome sequencing data. This transcriptional impairment translated to enzymatic dysfunction, with activity assays demonstrating severely compromised NR and NIR activities in the mutant (Fig. S3). Notably, nitrate reductase activity was particularly dependent on *BdAreA* regulation. Together, these data establish *BdAreA* as a master transcriptional activator that coordinately controls both the expression and functional output of the core nitrate assimilation machinery during nitrogen stress, providing mechanistic insight into the nitrate utilization defects of the mutant.

### *BdAreA* negatively regulates the expression of *BdHog1*

Transcriptomic profiling of *BdAreA* knockout strains in *B. dothidea* revealed widespread gene expression changes under standard YEPD culture conditions (Fig. S3B). KEGG pathway analysis identified significant alterations in MAPK signaling components, prompting focused investigation of *BdHog1,* a central regulator of stress responses. Quantitative validation by RT-qPCR (Fig. 6C) showed that while *BdAreA* deletion caused only a marginal (non-significant) reduction in *BdHog1* expression under nutrient-replete conditions, nitrogen starvation triggered dramatic transcriptional derepression, with *BdHog1* levels becoming significantly elevated in knockout strains compared to the wild-type strain (p < 0.05). This inverse relationship establishes *BdAreA* as a negative regulator of *BdHog1* specifically during nitrogen stress. Interestingly, reciprocal analysis of *BdAreA* expression in Δ*BdHog1* backgrounds revealed a more complex regulatory interaction (Fig. 6D). During nitrogen starvation, *BdAreA* transcripts in Δ*BdHog1* displayed a distinct biphasic pattern—initial suppression followed by robust induction. The underlying mechanism for this phenomenon remains unknown and requires further investigation. Collectively, these findings reveal a corresponding relationship between the two core signaling pathways, where *BdAreA* participates in the fungal response to nitrogen availability through its regulation of *BdHog1*.

### *BdAreA* mediates the dephosphorylation of *BdHog1*

Phosphorylation-dependent activation constitutes the core regulatory mechanism governing Hog1-type MAP kinases. Expanding from our qRT-PCR data revealing *BdAreA*-mediated transcriptional regulation of *BdHog1* during nitrogen stress, we systematically investigated protein-level modifications. Comparative analysis of Hog1 phosphorylation by Western blot revealed significant differences between WT and Δ*BdAreA* genetic backgrounds (Fig. 6E). Wild-type strains exhibited rapid Hog1 dephosphorylation upon acute nitrogen deprivation, showing a 60% (p < 0.05) reduction in phospho-Hog1 levels within 1 h compared to nutrient-sufficient conditions. This stress-responsive regulation was entirely abrogated in Δ*BdAreA*, which maintained constitutive Hog1 phosphorylation irrespective of nitrogen availability. Strikingly, *BdAreA*'s regulatory impact persisted under basal conditions, as Δ*BdAreA* displayed markedly diminished Hog1 phosphorylation even in YEPD medium. This reveals that under nitrogen starvation conditions, the deletion of *BdAreA* affects the phosphorylation status of BdHog1, although the precise mechanism (direct or indirect) remains to be elucidated.

## Discussion

Fungal GATA-type transcription factors are well-established global regulators that coordinate essential biological processes, including hyphal development, metabolic regulation, stress adaptation, and virulence. The functional versatility of these proteins is exemplified by Nsd1-mediated control of reproductive development [19–21], Ams2-dependent regulation of chromatin dynamics and chromosome segregation [22, 23], and Sre-mediated iron homeostasis through siderophore biosynthesis [24, 25]. Among these, AreA stands out as the master regulator of nitrogen metabolism [9], while Sfh1 contributes to genome stability through chromatin repair [26]. Notably, while the core function of AreA in nitrogen regulation is evolutionarily conserved, its ancillary roles display remarkable species-specific divergence.

*B. dothidea* represents an important phytopathogenic fungus [27], and we conducted functional characterization of *BdAreA* using targeted gene replacement. Comprehensive phenotypic analysis revealed *BdAreA*'s pleiotropic roles in vegetative growth, conidiogenesis and pathogenic development. Moreover, this study is the first to reveal that the absence of BdAreA significantly enhances the strain's sensitivity to both oxidative stress and phytoalexins in *B. dothidea*. Additionally, RNA-Seq analysis indicated that *BdAreA* regulates peroxisome biosynthesis, which may explain the hypersensitivity of Δ*BdAreA* to H₂O₂ stress (Fig. S3B). Collectively, these findings suggest that this heightened sensitivity may also underlie the reduced virulence observed in the knockout mutant. This important finding not only expands our understanding of the functional diversity of *BdAreA*, but more critically, provides the first evidence that this transcription factor plays a key role in regulating the fungal response to external stress. The results offer new theoretical insights for further elucidating the molecular mechanisms of fungal fungicide resistance.

The GATA-type transcription factor AreA is recognized as the master regulator of nitrogen catabolite repression (NCR), which activates the expression of NCR-repressed genes to facilitate fungal utilization of alternative nitrogen sources (nitrate, peptides, purines, proteins, and free amino acids) when preferred nitrogen sources (e.g., glutamine or ammonium) are limited [12]. Our findings demonstrate that *BdAreA* deletion in *B. dothidea* completely abolished nitrate assimilation capacity, phenocopying the conserved AreA-null phenotype previously reported in *F. graminearum* [9]. Mechanistically, we found that *BdAreA* coordinately upregulates both the transcript levels and enzymatic activities of nitrate reductase (BdNR) and nitrite reductase (BdNIR), thereby governing nitrate metabolic flux. Notably, while the nitrogen metabolic repressor NmrA has been shown to interact with and inhibit AreA activity in *G. fujikuroi* specifically under glutamine-replete conditions [28], our yeast two-hybrid assays failed to detect any physical interaction between BdNmrA and BdAreA (Fig. S4), suggesting potential divergence in regulatory mechanisms among fungal species.

The MAPK signaling pathway, a highly conserved eukaryotic signal transduction cascade, transmits extracellular stimuli through kinase phosphorylation cascades [29, 30]. By combining quantitative RT-qPCR with phosphoproteomic profiling, we revealed a regulatory relationship between *BdAreA* and *BdHog1*, which appears to be mediated by dynamic changes in the phosphorylation status of *BdHog1*. Furthermore, the attenuated virulence observed in Δ*BdAreA* may also be associated with this regulatory mechanism. Importantly, this study demonstrates that *BdAreA* acts as a key factor bridging the MAPK-HOG signaling cascade and nitrogen metabolism in *B. dothidea*, although the detailed molecular mechanisms underlying this cross-pathway regulation require further investigation.

In conclusion, our findings confirm that *BdAreA* in *B. dothidea* shares functional conservation with AreA orthologs in other filamentous fungi. Moreover, the discovery of the regulatory relationship between *BdAreA* and *BdHog1* provides novel insights into the molecular mechanisms governing *B. dothidea* pathogenicity and establishes a theoretical foundation for developing targeted fungicidal strategies. This study holds significant implications for achieving sustainable and eco-friendly control of *B. dothidea* in apple production.

## Materials and Methods

### Fungal strains and culture conditions

The wild-type strain LW03 of *B. dothidea* (the causal agent of apple ring rot), isolated and maintained by our laboratory, was cultured on potato dextrose agar (PDA; 200 g potato, 20 g dextrose, 15 g agar, and 1 L ddH₂O) at 25 °C in the dark. All transformants used in this study were generated as part of this research.

### Quantitative real-time PCR (RT-qPCR) analysis

This study systematically analyzed the expression levels of target genes using real-time quantitative PCR (RT-qPCR). Fungal strains were first cultured in modified nitrogen-deficient Czapek's liquid medium (denoted as NS throughout the study) (0.5 g KCl, 1 g KH₂PO₄, 0.5 g MgSO₄·7H₂O, 0.01 g FeSO₄·7H₂O, 30 g sucrose, adjusted to 1 L with ddH₂O). Total RNA was extracted from mycelia collected at different time points using the Spin Column Fungal Total RNA Purification Kit (Sangon Biotech, Shanghai). Subsequently, 1 μg of total RNA was reverse-transcribed into cDNA using the HiScript II 1st-Strand cDNA Synthesis Kit (with gDNA removal function, Vazyme). RT-qPCR was performed using SYBR Master Mix (TaKaRa, Dalian) with the Actin gene as the internal reference. The experiment included two independent biological replicates, each with three technical replicates.

To systematically evaluate gene expression patterns, comparative analyses were performed across different culture media: PDA medium, fruit medium (200 g 'Fuji' apple fruit + 20 g agar, adjusted to 1 L with ddH₂O), and bark medium (100 g 'Fuji' apple bark + 20 g agar, adjusted to 1 L with ddH₂O). For pathogenesis-stage analysis, RNA was extracted from the infection frontier (disease-health junction) of apple fruits at different time points using the SteadyPure Plant RNA Extraction Kit (Accurate Biotechnology). Mycelia grown on PDA (0 h) served as the control. The relative expression level of *BdAreA* was calculated using the 2^−ΔΔCq^ method. All experimental procedures strictly followed the manufacturer’s protocols to ensure data reliability.

### Deletion and complementation construction

The genetic information of AreA was retrieved from the NCBI database. The *BdAreA* deletion vector was constructed using a double-joint PCR strategy, with the hygromycin B phosphotransferase (*HPH*) gene as the selectable marker. Target fragments were fused with the resistance gene via overlap PCR [31]. The knockout construct was then introduced into the wild-type strain LW03 through polyethylene glycol (PEG)-mediated protoplast transformation. Hygromycin-resistant deletion mutants were subsequently screened and verified by PCR amplification [32] using the following validation primers: external verification primers F: 5’-GAAGAGCAACTGTAACGAGG-3’, R: 5’-TCCTCCACCGCTGTCAATCT-3’ and quantitative PCR primers F: 5’-CAACCAACGACGATAAGAGG-3’, R: 5’-TGGCTCTGTTTCCTTATCGG-3’.

For complementation, the *BdAreA* target gene was ligated with a geneticin (G418) resistance cassette. The recombinant fragment was purified by PCR amplification and transformed into protoplasts of the Δ*BdAreA* mutant. ransformants were screened through PCR verification using the validation primers F: 5’-ATTCCACCGCCGCCTTCTAT-3’ and R: 5’-TCCTCCACCGCTGTCAATCT-3’, and subsequent sequencing conducted by TsingKe Biotechnology Co., Ltd. confirmed the successful replacement of the HPH marker, ultimately yielding stably inherited complementation strains.

The BdHog1 knockout mutant used in this study was previously generated by our laboratory. For detailed information on its construction, please refer to our previously published work [30].

### Asexual reproduction and pathogenicity assays

This study first examined the asexual reproduction capacity of WT, Δ*BdAreA* and Δ*BdAreA-C.* The specific protocol included inoculating each strain on carrot agar medium (200 g carrot, 20 g agar, adjusted to 1 L with ddH₂O) for 3 days, followed by scraping aerial hyphae with a sterile spatula and evenly spreading them with 0.25% Tween 60 solution. The cultures were then incubated under black light at 25 °C for 14 days, with periodic observation and documentation of fruiting body formation and conidiation.

For pathogenicity analysis, an artificial wound inoculation method was employed. Each strain was inoculated onto mature 'Fuji' apple fruits, twigs, and detached leaves, with blank PDA medium serving as the negative control. Inoculated materials were maintained in a 25 °C constant-temperature humidity chamber, and disease progression was observed at 5 and 7 days post-inoculation (dpi). Lesion characteristics were meticulously recorded and photographed [32]. To ensure statistical robustness, the experiment included three independent biological replicates, each comprising 10 parallel samples. Quantitative analysis was performed by measuring lesion diameters.

### Sensitivity assay

To evaluate fungal growth characteristics, the mycelial growth rates of the tested strains were measured on PDA plates amended with various compounds. Modified PDA plates containing the following stressors were prepared: osmotic stressors: 0.5 M and 1 M sorbitol, 1 M NaCl, 1 M KCl; cell wall stressors: 0.8 mg/mL Congo red (CR), 0.25 g/L SDS; oxidative stressors: 0.15% H_2_O_2_; secondary metabolites: 0.5 mg/mL BOA, 25 mg/L 2-AP, 0.028% t-BHP, 68.87 mg/L menadione. All plates were incubated at 25 °C for 3 days. Colony diameters were measured to assess strain sensitivity to each inhibitor, and inhibition rates were calculated for statistical analysis. The experiment included three independent biological replicates. Data were analyzed using one-way ANOVA with LSD post-hoc tests in SPSS to determine significant differences between treatment groups.

### Nitrogen source utilization assay

Fungal strains (wild-type, knockout mutant, and complemented strains) were cultured on NS solid medium and modified Czapek's media supplemented with different single nitrogen sources (10 mM NaNO_3_, NH_4_Cl, urea, or glutamine) at 25 °C for 3 days to observe growth phenotypes. Parallel liquid culture experiments were conducted by inoculating equivalent amounts of mycelia into corresponding liquid media, followed by incubation at 25 °C with 180 rpm shaking for 5 days. Mycelial dry weights were subsequently measured for quantitative analysis.

### Enzyme activity assay

Mycelia cultured in yeast extract peptone glucose medium (YEPD, 10 g of peptone, 3 g of yeast extract, 20 g of dextrose, and 1 L of ddH_2_O, pH = 6.7) and treated with NS medium for 1 h were ground into powder in liquid nitrogen. Subsequently, 0.1 g of the powder was homogenized in 1 mL of extraction buffer and incubated on ice for 30 min. The subsequent procedures were carried out following the manufacturer's instructions for the nitrate reductase (NR) and nitrite reductase (NIR) activity assay kits (Sangon Biotech, Shanghai).

### Transcriptome analysis

WT and Δ*BdAreA* were cultured in YEPD liquid medium for 24 h, flash-frozen in liquid nitrogen, and subsequently submitted to Novogene (Tianjin, China) for transcriptome sequencing analysis.

### Western blotting assays

We performed protein extraction and immunoblot analysis following established methods [4]. To evaluate Hog1 phosphorylation status, fungal strains were first grown in liquid YEPD medium at 25 °C with shaking (180 rpm) for 24 h before being transferred to nitrogen-depleted Czapek's medium for 1 h to induce nitrogen starvation. Phosphorylated Hog1 was detected using a phospho-specific p38 MAPK antibody (Thr180/Tyr182; Cell Signaling Technology), with total Hog1 protein levels monitored using a C-terminal anti-Hog1 antibody (Santa Cruz Biotechnology). All antibodies were employed in accordance with their respective manufacturers' protocols.

## Supplementary Information

Supplementary Material 1. Fig. S1 Construction of a phylogenetic tree of BdAreA as well as different GATA transcription factors from other species based on amino acid sequences. The phylogenetic tree was constructed by MEGA11. Program using the neighbor-joining method.

Supplementary Material 2. Fig. S2 Targeted deletion of BdAreA. A: Schematic representation of the BdAreA replacement strategy; B and C: identification of the BdAreA deletion mutants by PCR amplification, external verification primers and quantitative PCR primers; D: identification of the BdAreA complemented strains by PCR amplification. M: DL250 DNA molecular marker.

Supplementary Material 3. Fig. S3 Enzyme activity assay and transcriptome sequencing. A: The effect of BdAreA knockout on BdNR and BdNIR enzyme activity. YE: indicates mycelia cultured in YEPD liquid medium for 12 hours; NS: represents mycelia subjected to nitrogen starvation treatment for 1 hour; B: Enrichment analysis KEGG (metabolic pathway) differential gene (B) between wild-type and mutant ΔBdAreA. The error bars represent the standard deviation of three independent experiments; asterisks indicate significant differences between groups (**P<0.01; ***P<0.001; ****P<0.0001).

Supplementary Material 4. Fig. S4 Verification of the interaction between transcription factor BdNmrA and BdAreA. The combination of AD-T + 53-BD and AD-T + Lam-BD served as the positive and negative controls, respectively.

Supplementary Material 5. Fig. S5 Determination of sensitivity to stress factors of ΔBdAreA. A: Sensitivity assays of the strains to t-BHP and menadione; B: The mycelial inhibition rate of each strain. The error bars represent the standard deviation of three independent experiments; asterisks indicate significant differences between groups ("ns" indicates no significant difference; **P<0.05; ****P<0.0001).

## Data Availability

All primary data and materials supporting the findings of this manuscript are available to any qualified researcher for the purpose of reproducing or extending the analysis. Requests for data access should be directed to the corresponding author and will be fulfilled within a reasonable timeframe.

## Abbreviation

2-AP: 2-Aminoacetophenone
AreA: GATA-type transcription factor AreA
BOA: Benzoxazolin-2-one
CR: Congo red
Hog1: High-osmolarity glycerol response Protein 1
HPH: Hygromycin B phosphotransferase
NCBI: National center for biotechnology information
NIR: Nitrite reductase
NR: Nitrate reductase
NS: Nitrogen-deficient Czapek's liquid medium
PCR: Polymerase chain reaction
PDA: Potato dextrose agar
PEG: Polyethylene glycol
RT-qPCR: Real-time quantitative PCR
WT: Wild-type strain
YEPD: Yeast extract peptone glucose medium
