# Supplementary figures and images for: BdAreA regulates nitrogen metabolism, stress response, and virulence in Botryosphaeria dothidea
Source: Crop Health. 2026 Jan 22;4(1):2. doi: 10.1007/s44297-026-00065-8 (PMC12827830; doi:10.1007/s44297-026-00065-8)

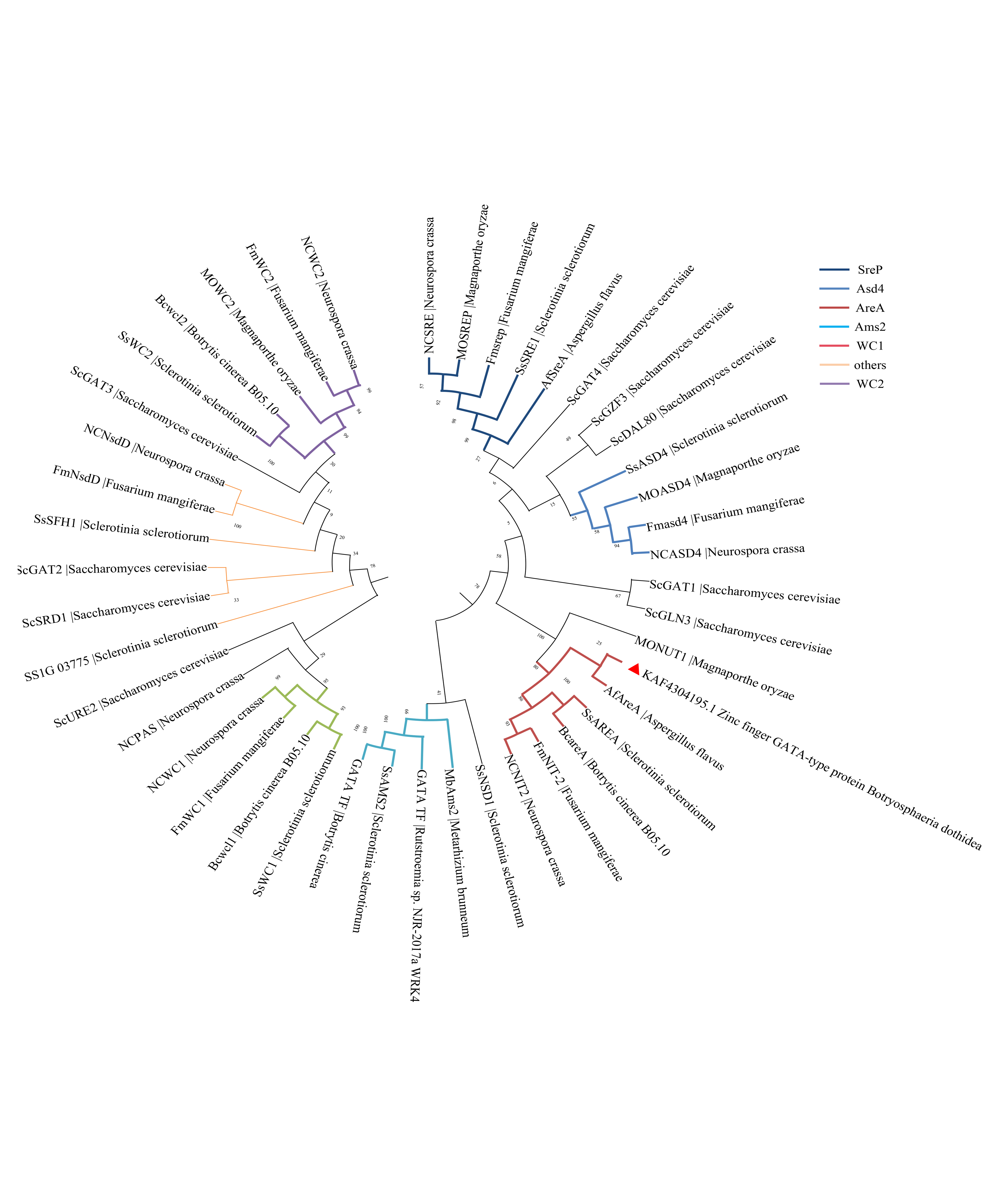

Supplement: Supplementary file 1 — Supplementary Material 1. Fig. S1 Construction of a phylogenetic tree of BdAreA as well as different GATA transcription factors from other species based on amino acid sequences. The phylogenetic tree was constructed by MEGA11. Program using the neighbor-joining method. [file 44297_2026_65_MOESM1_ESM.png]

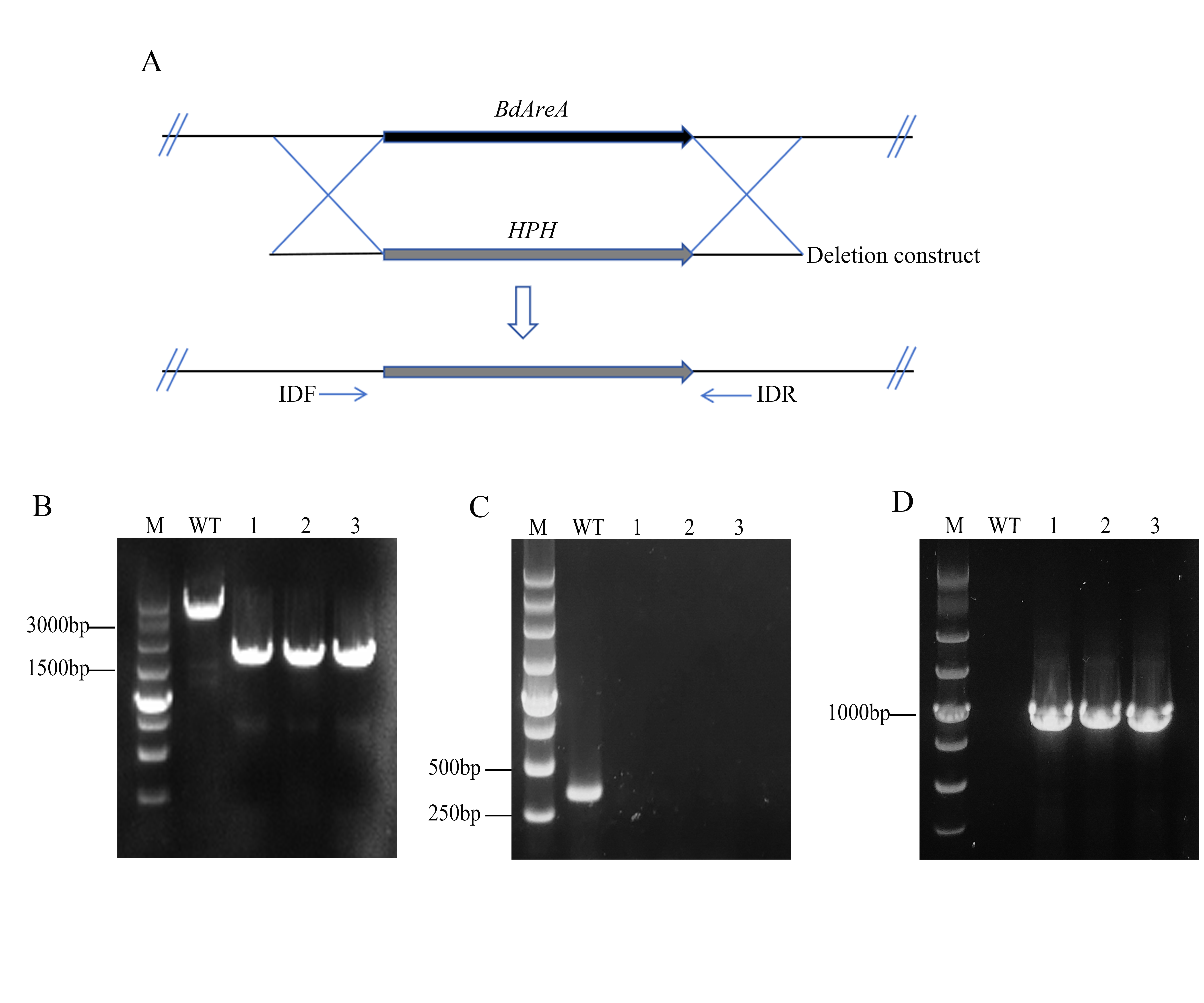

Supplement: Supplementary file 2 — Supplementary Material 2. Fig. S2 Targeted deletion of BdAreA. A: Schematic representation of the BdAreA replacement strategy; B and C: identification of the BdAreA deletion mutants by PCR amplification, external verification primers and quantitative PCR primers; D: identification of the BdAreA complemented strains by PCR amplification. M: DL250 DNA molecular marker. [file 44297_2026_65_MOESM2_ESM.png]

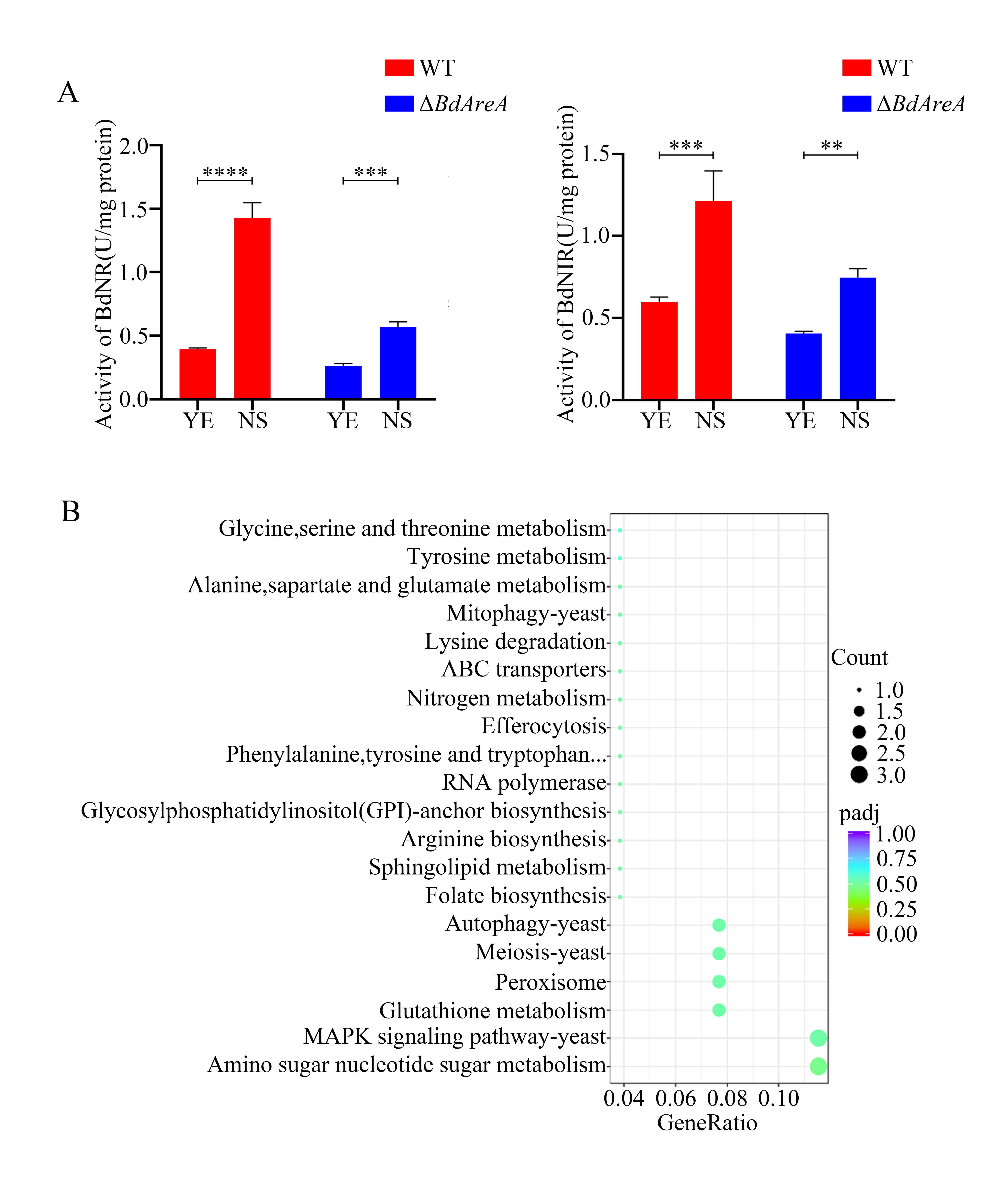

Supplement: Supplementary file 3 — Supplementary Material 3. Fig. S3 Enzyme activity assay and transcriptome sequencing. A: The effect of BdAreA knockout on BdNR and BdNIR enzyme activity. YE: indicates mycelia cultured in YEPD liquid medium for 12 hours; NS: represents mycelia subjected to nitrogen starvation treatment for 1 hour; B: Enrichment analysis KEGG (metabolic pathway) differential gene (B) between wild-type and mutant ΔBdAreA. The error bars represent the standard deviation of three independent experiments; asterisks indicate significant differences between groups (**P<0.01; ***P<0.001; ****P<0.0001). [file 44297_2026_65_MOESM3_ESM.png]

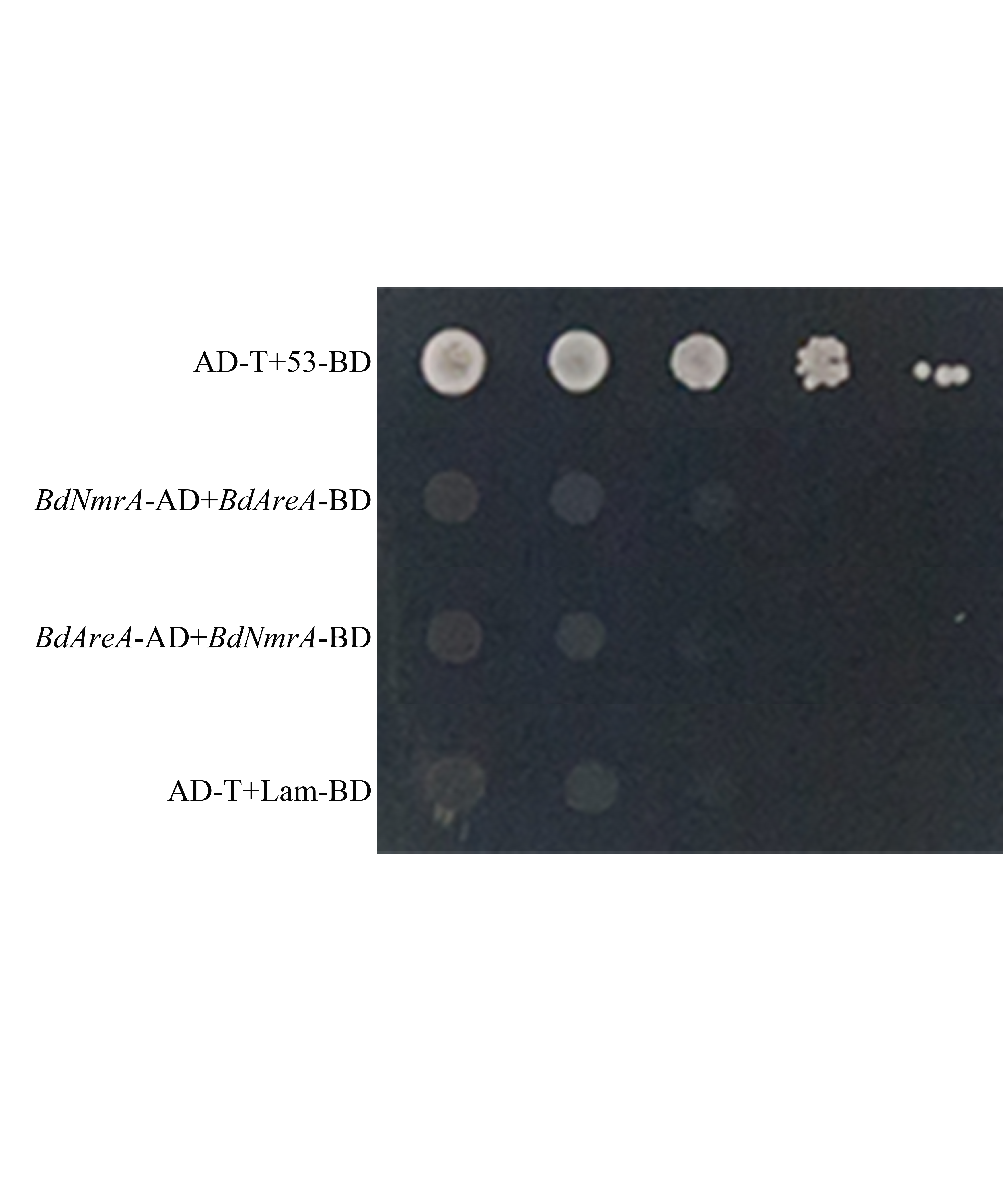

Supplement: Supplementary file 4 — Supplementary Material 4. Fig. S4 Verification of the interaction between transcription factor BdNmrA and BdAreA. The combination of AD-T + 53-BD and AD-T + Lam-BD served as the positive and negative controls, respectively. [file 44297_2026_65_MOESM4_ESM.png]

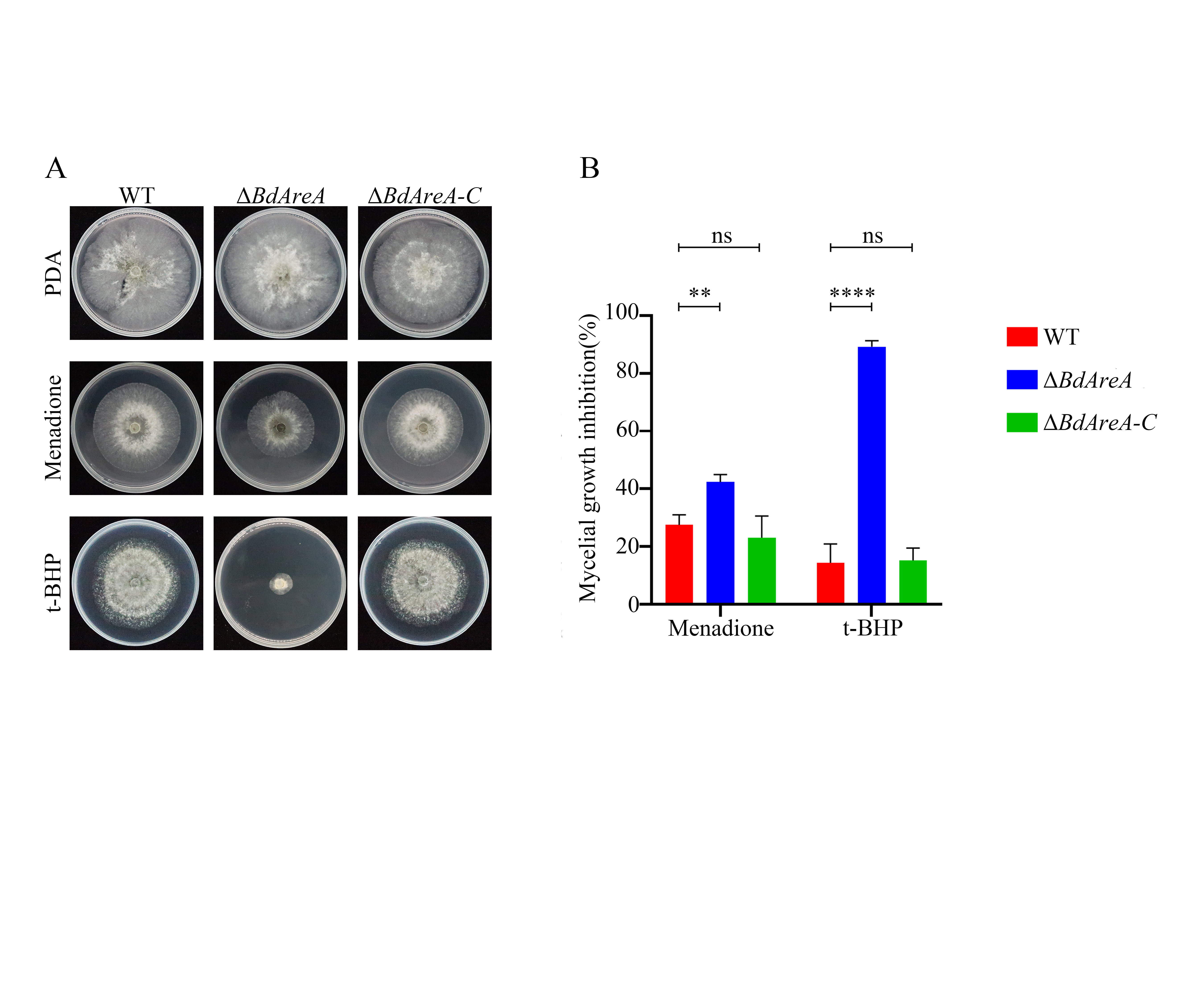

Supplement: Supplementary file 5 — Supplementary Material 5. Fig. S5 Determination of sensitivity to stress factors of ΔBdAreA. A: Sensitivity assays of the strains to t-BHP and menadione; B: The mycelial inhibition rate of each strain. The error bars represent the standard deviation of three independent experiments; asterisks indicate significant differences between groups ("ns" indicates no significant difference; **P<0.05; ****P<0.0001). [file 44297_2026_65_MOESM5_ESM.png]
